# Nanotechnology in Cement-Based Materials: A Review of Durability, Modeling, and Advanced Characterization

**DOI:** 10.3390/nano9091213

**Published:** 2019-08-28

**Authors:** Sen Du, Junliang Wu, Othman AlShareedah, Xianming Shi

**Affiliations:** Department of Civil and Environmental Engineering, Washington State University, Pullman, WA 99164-2910, USA

**Keywords:** cement-based material, nanomaterial, physical deterioration, chemical deterioration, rebar corrosion, nanomodeling, advanced characterization

## Abstract

In the context of increasing applications of various nanomaterials in construction, this work reviews the renewed knowledge of nanotechnology in cement-based materials, focusing on the relevant papers published over the last decade. The addition of nanomaterials in cement-based materials, associated with their dispersion in cement composites, is explored to evaluate their effects on the resistance of cement-based materials to physical deteriorations, chemical deteriorations, and rebar corrosion. This review also examines the proposed nanoscale modeling of interactions between admixed nanomaterials and cement hydration products. At last, the recent progress of advanced characterization that employs techniques to characterize the properties of cement-based materials at the nanoscale is summarized.

## 1. Introduction

In the construction industry, cement paste, mortar, and concrete are the most commonly used materials, due to their easiness to fabricate, low expenditure, good performance, and versatile applications. However, the drawbacks of these cement-based materials (CBMS), such as low tensile strength, susceptibility to cracking (if tensile stress induced by deterioration exceeds tensile strength), and likelihood of sudden failure (due to their brittle nature), have led to multiple pathways of degradation in the technical properties of CBMS and high costs of repairing them [1]. As the main cement hydration product, calcium–silicate–hydrates (C–S–H) is the main phase that combines aggregates together, forming the strength and other macroscopic engineering properties of CBMS. The size of the basic structural unit of C–S–H lies in the nanometer range. Understanding the characteristics of C–S–H at the nanoscale should facilitate the efficient manipulation of the physicochemical nature of CBMS [2]. Therefore, the application of nanomaterials in CBMS, which have remarkable influence on the modification of C–S–H, is one of the best ways to tackle the aforementioned concerns over CBMS.

Recently, the use of nanomaterials to improve the mechanical properties and durability performances of CBMS has received considerable attention. It is believed that the improvement effects by admixing nanomaterials in CBMS are mainly through two approaches: (1) acting as a superb filler and (2) involving in the cement hydration [3,4]. The photocatalytic effect of nanotitanium is also usually reported in the literature, which endows the surface of CBMS with a self-cleaning ability in the presence of ultraviolet light [5]. However, the functionalization of cement composites by admixing nanomaterials is outside the scope of this review. This work focuses on the enhancement of the resistance of CBMS to physical deteriorations, chemical deteriorations, and rebar corrosion by applying nanotechnology. Despite the fact that nanomaterials may cause deficiencies in some properties of CBMS, such as reduced workability due to the high water demand of these ultrafine particles, their beneficial effects on technical properties of CBMS have been clearly demonstrated [4]. The most commonly used nanomaterials are nanoscale spherical particles (nano-SiO_2_, TiO_2_, Al_2_O_3_, Fe_2_O_3_ etc.), nanotubes and fibers (carbon nanotubes and carbon nanofibers), and nanoplatelets (nanoclays, graphene, and graphite oxide) [6,7,8].

From the review of literature, there is an increasing usage of nanotechnology in cement paste, mortar, and concrete, including both increased availability of newly developed nanomaterials and greater use of nanomaterial-modified cement composites [5]. Nanotechnology in the CBMS mainly includes two directions: developing new products which are engineered at the nanometer scale for the concrete industry; characterizing and understanding the materials at the nano- (and sometimes micro-)scale through the use of atomic modeling and advanced characterization techniques [2,9,10]. The application of nanotechnology in CBMS in the last decade has seen increased and more-informed utilization of nanomaterials. Given the enormous amount of work done in this field, a comprehensive review on this topic would require much more space than a single article. Moreover, there may exist a substantial difference between the laboratory studies from academic literature and the realities of producing the cement composites used in the field [5]. In the construction industry, the real concern from practitioners and decision-makers is the cost-effectiveness of admixing nanomaterials in improving the technical properties of CBMS. 

**Figure 1:**
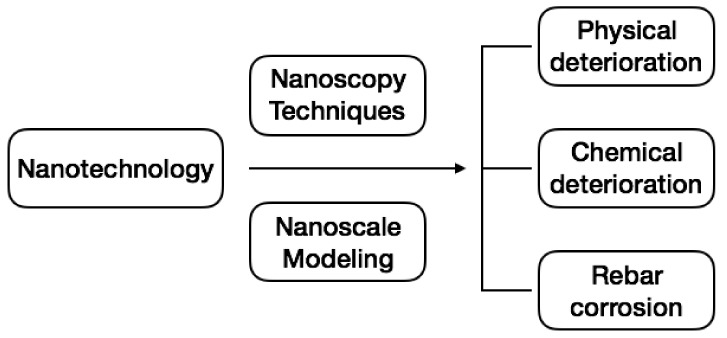
Overview of the sections in this review article, mainly based on the relevant papers published in the last decade.

In this context, this review begins by considering the dispersion of nanomaterials in CBMS and what measures can be taken to improve their dispersion abilities, which is the principal factor that affects their successful applications in cement composites. Then, we present a summary on the effects of nanomaterials on the improvement of CBMS when subjected to physical deteriorations (shrinkage, thermal cracking, freeze–thaw damage, and abrasion), chemical deteriorations (sulfate attack, acid attack, alkali-aggregate reaction, and thermal degradation) and corrosion (for steel reinforcement concrete). At last, we review the underlying mechanisms for these improvements; mainly through two approaches, i.e., modeling the nanoscale interactions between C–S–H and admixed nanomaterials and applying advanced nanoscopy characterization techniques that are most frequently used by researchers (Figure 1). 

## 2. Dispersion of Nanomaterials in CBMS

The type and dosage of nanomaterials and the associated nanotechnology investigated in academic literature may differ from the realities in practice [5]. A main issue that hinders the application of nanomaterials in CBMS is their dispersion, which is crucial for the effectiveness of unlocking the potential of the admixed nanomaterials and improving the properties of the cement composites. Thus far, there has not been a method that is widely accepted to quantify the dispersion level of nanomaterials in the cement matrix. Most of the studies adopt an indirect method, which measures the mechanical properties of CBMS to reflect if there is any agglomeration of nanomaterials in the cement composites. This is based on the fact that the poorly dispersed nanomaterials present in the matrix tend to agglomerate, forming flocs, clusters, or bundles due to the attractive van der Waals force, some of which act as defects in the matrix and thus degrade the mechanical properties of cement composites [11,12,13]. For instance, carbon nanotube (CNT), featuring a hydrophobic nature, is difficult to disperse uniformly in cement composites [5,11]. A study by Rocha and Ludvig [11] demonstrated that there was an optimal dosage of 0.05 wt% for CNT to be incorporated in the cement matrix, beyond which aggregation of CNT may occur, resulting in a reduced gain in the mechanical properties of cement paste. In this study, CNT was not functionalized by surface treatment, but dispersed in a non-aqueous isopropanol media with the aid of ultrasonication for 2 h.

**Figure 2:**
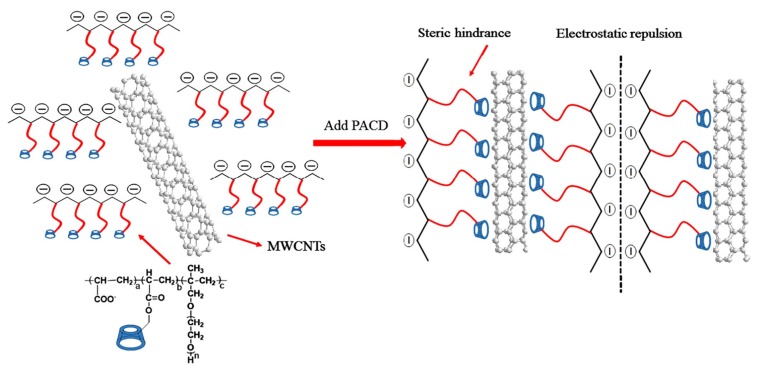
Dispersion mechanism for multiwalled carbon nanotube (MWCNT) with cyclodextrin-modified polycarboxylate superplasticizer (PACD). Reproduced from [13], with permission from John Wiley and Sons, 2019.

Generally, there are two methods that functionalize CNT to promote its dispersion and prevent its aggregation, namely, covalent and noncovalent methods [13,14]. The covalent functionalization method involves inducing chemical functional groups on the side walls of CNT, thereby improving the adherence of CNT to the cement matrix. As a result, a good dispersion of CNT in cement composites can be achieved. However, some authors [15] have suggested that the induced functional groups can reduce the mechanical strengths of CNT, thus negatively affecting the performance of the composites. As such, the noncovalent functionalization method that treats the surface of CNT with surfactant (e.g., superplasticizer) or uses other nanomaterials along with CNT has become popular [14,16,17]. Meng et al. [13] have demonstrated that the polycarboxylate ether (PCE) superplasticizer can improve the dispersibility of CNT and reduce its aggregative tendency in water. In their study, one specific superplasticizer, namely cyclodextrin-modified polycarboxylate superplasticizer (PACD), was used as surfactant to disperse multiwalled CNT (MWCNT). Figure 2 illustrates the dispersion mechanism of MWCNT with the aid of PACD, which exhibits a dual effect of steric hindrance and electrostatic repulsion that both preclude CNTs from approaching each other. By using the sol–gel method, Stynoski et al. [18] functionalized CNT with nanosilica and reported that the dispersion stability was significantly improved.

Superplasticizer has been widely used as dispersing agent for nanomaterials in CBMS [12,19,20,21]. Pérez-Nicolás et al. [12] analyzed the zeta potential of nanomaterial dispersions and reported that a specific type of superplasticizer (naphthalene-based) exhibited good capability to disperse nano-TiO_2_ in water through mechanically stirring for 20 min. The zeta potential test indicated that the largest negative value can be observed for the combination of this specific superplasticizer and nano-TiO_2_, resulting from the electrostatic repulsions. Qian and Schutter [20] studied the compatibility between PCE superplasticizer and nanoclay. They found that there was an optimal dosage of PCE which showed a beneficial effect on the dispersion of nanoclay in the cement paste and prevented the occurrence of agglomeration given a constant amount of nanoclay. In terms of the thixotropy and yield stress of the cement paste, the combination of 0.2 wt% PCE and 0.5 wt% nanoclay exhibited the best performance. 

In addition to using superplasticizer as a surfactant to disperse nanomaterials in CBMS, ultrasonication is another method that is usually used to improve the distribution of the nanomaterials [22,23,24]. By comparing the effect of mechanical stirring and ultrasonication on the dispersion of nano-TiO_2_ in cement mixes, Yousefi et al. [22] evaluated the photocatalytic properties of different cement samples. Their study showed that a high level of agglomeration of nano-TiO_2_ would be observed if no ultrasonication was applied. Moreover, the photocatalytic properties of the cement sample were better when the nano-TiO_2_ was dispersed by ultrasound.

## 3. Nanotechnology for CBMS to Overcome Physical Deteriorations

In practice, the durability of CBMS can be affected by vulnerability to damages induced by external loading in combination with environmental effects, such as abrasive/erosive actions, moisture and temperature fluctuations, and freeze–thaw cycles [25]. Therefore, it is imperative to make modifications in the current concrete technology for making the concrete a more durable and sustainable product, which can perform more cost-effectively and reliably [26]. Nanomaterials with an ultrafine size show unique physical and chemical characteristics, and their presence in fresh CBMS can induce properties that are different from those of the conventional cement composites [27]. In fact, nanomaterials can be employed in the CBMS for significantly enhancing the resistance to crack initiation and restraining the crack propagation, which are the main results from the physical distresses or damages of CBMS, such as shrinkage, freeze–thaw damage, and abrasion [28]. This section explores the effects of admixing nanomaterials in CBMS on mitigating the physical degradations of CBMS.

### 3.1. Shrinkage

If shrinkage is restrained in concrete (or other cementitious composites), then the restrained shrinkage will create tensile stress, which can cause cracks in concrete if the local stress exceeds the local strength [29]. Usually, the total shrinkage of CBMS measured can be the joint outcome of drying shrinkage, plastic shrinkage, and autogenous shrinkage. The total shrinkage of CBMS can be reduced by the addition of nano-TiO_2_, nano-CaCO_3_, nanosilica, and carbon nanofiber [30,31,32]. Yang et al. [30] analyzed the effect of incorporating nano-TiO_2_ on the total shrinkage of the alkali activated slag paste. In their study, the accelerated cement hydration process and a denser microstructure were observed due to the admixed nano-TiO_2_, contributing to the reduction of the amount of mesopores (featuring a size of 1.25–25 nm). Hence, nano-TiO_2_ remarkably reduced the total shrinkage of the paste, which is considered a reflection of the amount of mesopores. Note that nano-TiO_2_ mainly works through the nucleation effect, i.e., providing nucleation sites for the accumulation of hydration products and thus regulating the hydration of cement. Similar conclusions were also drawn by Liu et al. [31], who compared the early age (12 h) shrinkage between a cement paste containing nano-CaCO_3_ and the reference paste and suggested that there was an optimal content for the nano-CaCO_3_. At the dosage of 1 wt%, the addition of nano-CaCO_3_ facilitates the early hydration of the cement, thus exhibiting a reduction effect on the early age shrinkage.

**Figure 3:**
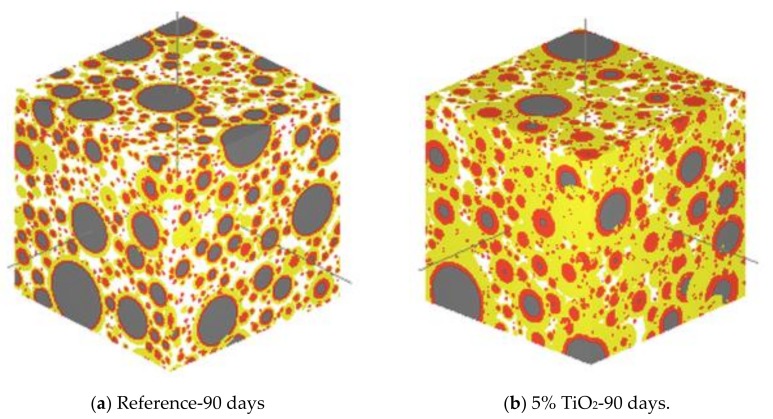
Formation of the microstructures in the cementitious material with or without nano-TiO_2_ (grey part: Unreacted particles; red part: Inner products; yellow part: Outer products; white part: Pores). Reproduced from [33], with permission from Elsevier, 2019.

**Figure 4:**
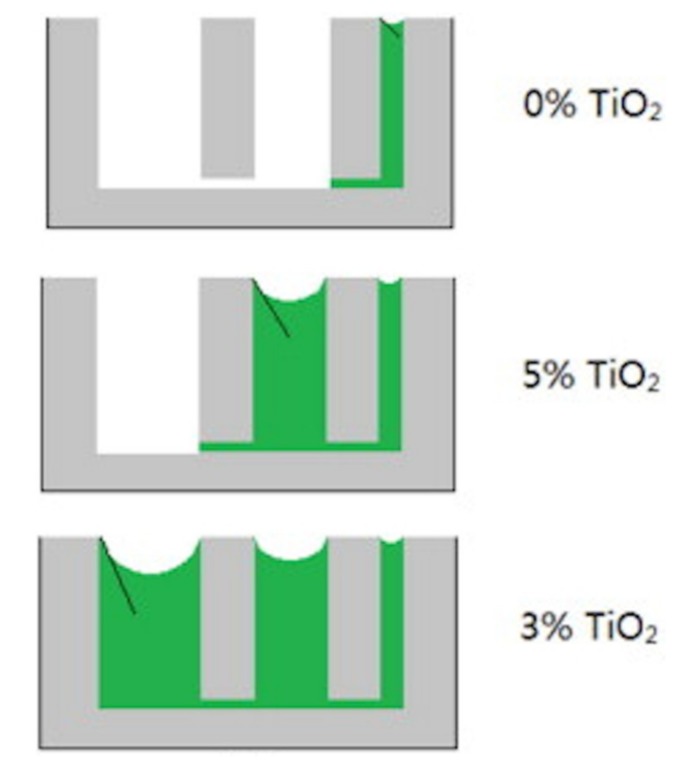
The sketch of the filling of water in the capillary pores before and after the addition of nano-TiO_2_ in the cement composite. Reproduced from [34], with permission from Elsevier, 2019.

Drying shrinkage occurs as concrete dries. In producing the concrete, the mixing water is usually added beyond the amount required for cement hydration in order to endow concrete with desirable workability for construction. When the concrete is exposed to ambient conditions, the excess water will evaporate and cause drying shrinkage [29]. From the literature, there is a contradictory conclusion about the effect of adding nanomaterials on the drying shrinkage of CBMS. Some nanomaterials, including nano-TiO_2_, synthetic nanofiber, and graphene oxide (GO), could reduce the drying shrinkage of cement composites [29,33,34,35]. Studies undertaken by Duan et al. [33] and Zhang et al. [34] have shown that the admixing of nano-TiO_2_ into CBMS can mitigate the drying shrinkage by forming a compact microstructure with less cracks and reducing water loss through the pore-refining effect and the hydrophilicity-increasing effect, as indicated in Figure 3 and Figure 4, respectively. Figure 3 shows that the amount of unreacted phases is reduced, whereas the size and amount of the hydration products are increased after 5 wt% nano-TiO_2_ is admixed into the composites. The decreased porosity and denser microstructure can also be observed, resulting in the improved resistance to drying shrinkage. Moreover, more capillary pores, especially those with a relatively large size, can be filled with water in the cement paste containing an optimal content of 3 wt% nano-TiO_2_, as shown in Figure 4. As a result, the water loss is reduced due to the increased hydrophilicity of the cement paste, which thereby mitigates the drying shrinkage. Lee and Won [29] have reported that the structural nanosynthetic fiber would lower the drying shrinkage by controlling the initial cracking of cement composites [29]. The effect of GO, which is a graphene-based nanomaterial, on the drying shrinkage of the cement paste has been investigated by Lu et al. [35]. Their study suggested that GO could mitigate the drying shrinkage of cement composites through the densified microstructures and reduced amount of capillary pores.

**Figure 5:**
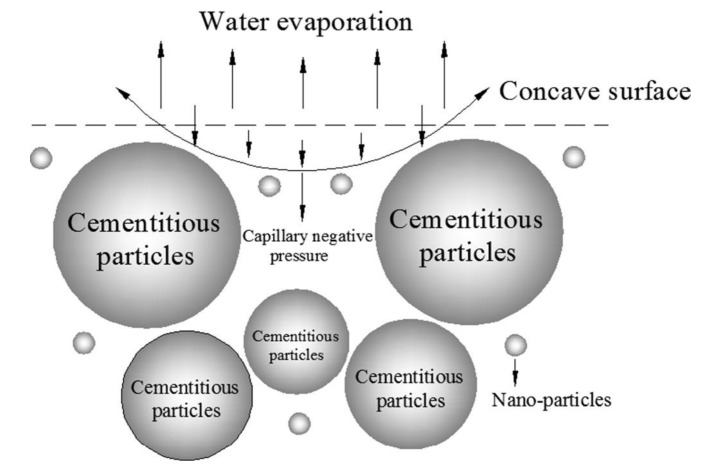
Force simulation of air-liquid meniscus. Reproduced from [36], with permission from Elsevier, 2019.

However, Gao et al. [36] reported that nano-SiO_2_ and nano silica carbide can aggravate the drying shrinkage when admixed in cement composites. This is mainly due to the water adsorption effect of these nanoparticles, which could absorb free water from the capillary pores in concrete. Figure 5 shows the continuous evaporation of surface water from the concrete containing nanoparticles after stopping the wet curing. Hence, the drying shrinkage of concrete was accelerated resulting from the increasing difference in the relative humidity between the concrete surface and its interior.

The autogenous shrinkage of CBMS usually occurs as a result of self-desiccation during the hydration process, which is mainly generated at early ages and proportional to the amount of the fine pores [37]. For high performance concrete, autogenous shrinkage becomes more prominent and is a dominant factor for the cracking control [38]. Cement composites reinforced with nanomaterials, such as CNT, nano-MgO, nano smectite based clay, exhibit lower autogenous shrinkage compared with those without nanomaterials [37,38,39]. Konsta-Gdouto et al. [37] investigated the effect of the dispersion of CNT on the autogenous shrinkage and indicated that the admixed CNT can decrease the porosity of the cementitious matrix. Their study revealed that the volume fraction of the fine pores, whose size in diameter is less than 20 nm, was apparently reduced by the addition of fine CNT. There was a close relationship between the number of fine pores and the autogenous shrinkage of the cement paste, both of which were reduced by the admixed CNTs. Polat et al. [39] analyzed the effect of admixed nano-MgO on the autogenous shrinkage of cement paste and reported that the autogenous shrinkage of the cement composite can be compensated by the expansion effect of nano-MgO, which can react with water to form expansive products. Similar conclusions were also drawn in another study [38], in which the admixed nano-MgO exhibited long-term expansive effects due to its slow reaction rate in concrete.

### 3.2. Freeze–Thaw Damage

Damage due to the freeze–thaw cycles is one of the major deterioration pathways of concrete in cold climates [40]. There are several main theories that are being widely accepted for explaining the freeze–thaw damage of cement composites, including the hydraulic pressure theory, crystallization pressure theory, and osmotic pressure theory [41]. Generally, the frost damage of CBMS results from the pressure induced by the volume increase associated with water turning from liquid phase to solid phase and by the water migration in the capillary pores [42]. The pressure will introduce stress in the internal microstructure of the concrete, which causes cracking if it exceeds the local strength of the concrete.

With the addition of nanomaterials, including nanosilica, nanoalumina, nanokaolinite clay, and GO, better resistance to freeze–thaw damage (i.e., frost damage) of CBMS can be observed, attributable to the denser and more compacted microstructure [40,41,42,43,44,45]. Gonzalez et al. [42] investigated the effect of admixing nanosilica on the damage of concrete subjected to freeze–thaw cycles. This study showed that the nanosilica in the concrete would act as a supplementary cementitious material (SCM), which can react with portlandite (a main product of cement hydration) to form additional C–S–H. As a result, the paste and the interface transition zone (ITZ) between the aggregate and paste were both improved. In addition, a refined pore structure in concrete was observed, and this translates to a limited intrusion of water and thus less water available for participating in the freeze–thaw damage. Therefore, the incorporation of nanosilica in concrete was able to reduce the frost damage. Similar conclusions were also drawn by Quercia et al. [43], who analyzed the effect of the nanosilica addition on the durability performance of concrete and demonstrated that the incorporation of 3.8 wt% nanosilica improved all durability indicators, including the freeze–thaw resistance. In addition, the highly stiff and small-size C–S–H gel generated due to the pozzolanic activity of nanosilica contributes to the densification of the microstructure and thus the improved frost resistance. Admixing GO in cement composites, Mohammed et al. [45] tested the weight loss of samples after exposure to 540 freeze–thaw cycles. The nitrogen absorption test uncovered that the GO in the cementitious matrix mainly exhibited the modification effect of the pore structures, which remarkably reduced the amount of mesopores. Because water freezes more difficultly in small pores than in large pores, this caused a weight loss of only 0.25% after 540 cycles in the samples containing GO (vs. 0.8% for the control samples).

### 3.3. Abrasion/Erosion

Abrasion resistance is one of the key considerations for concrete, especially when concrete is exposed to abrasive forces in some specific applications, such as on the pavement surface, in the dam structure, or in the bridge footing [46]. The abrasion of concrete is usually caused by the scraping, rubbing, skidding or sliding of objects on the surface of concrete [47]. Nanomaterials, such as nano-TiO_2_, nano-SiO_2_, and nanosilica carbide, have been explored in various studies to enhance the resistance of concrete to abrasion [36,48]. Gao et al. [36] investigated the effect of nanosilica on the wear resistance of fly ash concrete and suggested that there was an optimal dosage for nanosilica to exhibit the beneficial influence. At the content of 2 wt%, nanosilica can reduce the wear loss of the concrete by 75%, when compared with the reference concrete. This is attributed to the pozzolanic reaction and microaggregate filling effect of the nanosilica, which improve the distribution of cementitious particles and the orientation degree of Ca(OH)_2_ in the cement composite. As a result, a denser texture formed, which leads to improved abrasion resistance. Similar conclusions were drawn in a study conducted by Li et al. [48], who reported the enhancement of abrasion resistance of concrete due to the addition of nano-TiO_2_. They interpreted the mechanism of this improvement as follows. If nano-TiO_2_ was dispersed in the cement matrix uniformly, the growth of hydration products would be controlled to accumulate on the nanoparticles due to their nucleation effect, resulting in a more homogeneous and compact cement matrix. As a result, the abrasion resistance of concrete was significantly improved by the admixed nano-TiO_2_.

## 4. Nanotechnology for CBMS to Overcome Chemical Deteriorations

When concrete is exposed to environmental conditions, it can be considerably affected by the chemical deteriorations during its service life [49]. This section presents the nanotechnology for CBMS subjected to chemical distresses or chemical pathways compromising their durability, including alkali–aggregate reactions, sulfate attack, acid attack, and thermal degradation [4,9].

### 4.1. Alkali-Aggregate Reactions

Alkali–aggregate reactions (AARs) occur when there are active phases in the aggregate and the alkalinity in the pore solution exceeds the threshold value. Generally, if the active phases are from amorphous silica aggregate, the reaction can be classified as an alkali–silica reaction (ASR). While the active phases are sourced from dolomitic limestone aggregate, they can cause the alkali–carbonate reaction (ACR) in concrete [50]. The products generated in AARs are typically expansive and will cause cracking in concrete if the tensile stress due to the expansion exceeds the local tensile strength of the concrete. From the literature, there are several factors, including water content, alkali content, aggregate activity, and temperature, which influence the degree of AARs [51]. Multiple studies indicate that the pozzolanic reaction, which consumes the Ca(OH)_2_ in concrete and thus reduces the alkalinity of the pore solution, can mitigate the effect of ASR [52,53,54]. As a result, the use of nanomaterials to accelerate the pozzolanic reaction will eventually contribute to the control of AARs. Aly et al. [55] used glass powder to replace cement (up to 40%) as the active phase in the cement matrix and tested the ASR of cement composites when 3 wt% nanosilica was admixed. The ASR test results indicated that no damaging effect can be detected. Moreover, the differential thermal analysis/thermogravimetric analysis and X-ray diffraction result demonstrated a reduction in the content of Ca(OH)_2_, which was attributed to the pozzolanic activity of nanosilica. As a result, the alkalinity in cement composites containing nanosilica was decreased to a value below the threshold, thereby preventing the occurrence of ASR. 

### 4.2. Sulfate Attack

In the field, sulfate attack can significantly undermine the durability of concrete structures. Generally, sulfate attack can form expansive compounds, as a result of a series of chemical reactions that occur between the aggressive sulfate ions and hydrates in the cement paste. The expansion effect of the sulfate attack in concrete results in cracking, strength loss, and softening of the cementitious matrix in the long term [49]. Some effective measures to mitigate the damages caused by sulfate attack include: reducing the permeability of concrete, lowering water-to-cementitious materials ratio, increasing the content of cement, or forming well-compacted microstructure [56]. 

Based on recent studies [57,58,59], nanosilica can improve the resistance of concrete to sulfate attack by utilizing its densification effect on the microstructure, which slows down the penetration of sulfate ions and water into the concrete. Ghafoori et al. [60] compared the effect of nanosilica and microsilica on the expansion of cement mortars under sulfate attack. Their study indicated that the addition of nanosilica significantly reduced the expansion of mortars. When nanosilica was dispersed uniformly in the matrix and no agglomeration was observed, it outperformed microsilica at the same dosage. Similar conclusions were also obtained by Arel and Thomas [61], who demonstrated that the addition of nanosilica reduced the expansion of cement mortar after 23 weeks of exposure to a sulfate environment, more so than the addition of microsilica. The reduced porosity and improved microstructure of samples containing nanosilica was attributed to both the nanofiller effect and the pozzolanic nature of nanosilica. When considering the relationship between the addition dosage and the capability of mitigating sulfate-induced expansion, nanosilica would be the most effective one when compared with microsilica, fly ash, or ground granulated blast furnace slag [49]. 

### 4.3. Acid Attack

**Figure 6:**
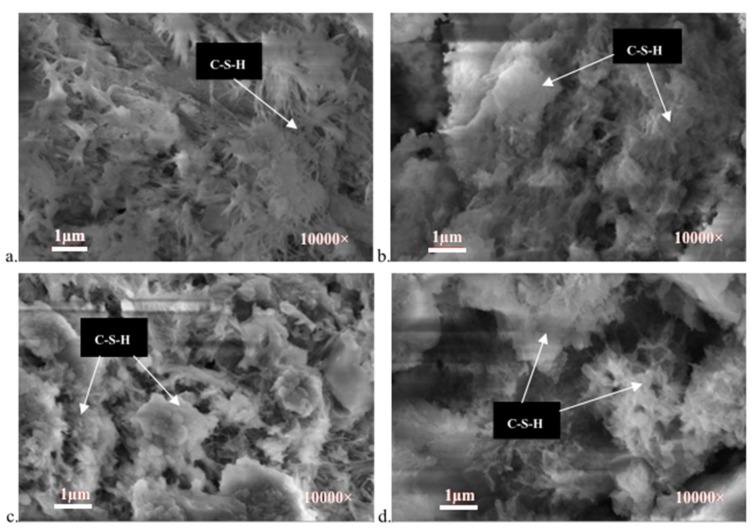
Microstructure of cement composite after acid solution corrosion: (**a**) No nanokaolinite clay (NKC) addition; (**b**) 1% NKC addition; (**c**) 3% NKC addition; (**d**) 5% NKC addition. Adapted from [67], with permission from Elsevier, 2016.

It is known that concrete deteriorates under acidic environments due to chemical attack. In the field, the acidic environments that can impose deleterious effects on the concrete components or structures mainly include ground water, industrial effluent, and acid rain [62]. Previous studies have discussed the mechanism of acid attack (i.e., acid corrosion) of CBMS. In the case of sulfuric acid, the deterioration of CBMS exposed to acid is mainly due to the presence of H^+^ and SO_4_^2−^ ions, which can lead to the dissolution of hydration products and the formation of expansive compounds [63,64,65,66]. The nanomaterial, such as calcined nanokaolinite clay (NKC), has been reported by Fan et al. [67] to improve the resistance of the cement mortar to acid solution exposure. In their study, cement mortar samples with or without NKC were submerged in a sulfate and nitric acid solution, which exhibited a pH value of 1.5. After exposure, the mass loss and residual compressive strength were measured. There was an optimal dosage of NKC that exhibited the beneficial influence on the improvement in the acid resistance of the cement mortar. At the dosage of 3 wt%, NKC reduced the mass loss and compressive strength loss of mortar samples by 19% and 17%, respectively, when compared with the control mortar samples without the addition of NKC after 60 days of exposure to the acid solution. Moreover, back-scattered electron microscopy images revealed that the C–S–H gel was decalcified due to the presence of H^+^ and an expansive CaSO_4_·2H_2_O crystal was formed due to the presence of SO_4_^2−^ in the cement mortar sample with no addition of NKC (Figure 6a). Figure 6c demonstrates that there was a greater amount of C–S–H gel in the sample with 3 wt% NKC addition, which was attributed to the filling effect and high activity of NKC, resulting in improved resistance to the acid attack.

### 4.4. Thermal Degradation

For structural concrete, the nonflammable nature and elevated-temperature resistance of CBMS can protect the reinforced rebar. However, exposure to an elevated temperature imposes a detrimental effect on the properties of CBMS, in which both chemical and physical transformations occur under thermal exposure. Nanomaterials, such as nanosilica, nanoalumina, nanoclay, CNT, GO, and graphene sulphonate nanosheet (GSNS), have been demonstrated to have the potential to impede the thermal degradation of CBMS [14,68,69,70,71,72,73,74,75,76,77]. A study aimed at using low-cost nanomaterials in the cement paste, has synthesized nanosilica from the rice husk ash and investigated its effect on the thermal resistance of cement pastes [69]. The study showed that there was an optimal dosage for the nanosilica which is beneficial in compensating the negative effect of elevated temperatures on the properties of the cement paste. Scanning electron microscopy (SEM) micrographs indicated that the inclusion of 1 wt% nanosilica in the cement paste exhibited the microfiller effect and pozzolanic activities, which were reflected in the dense and compact microstructure. As a result, the residual compressive strength of the cement paste fired at elevated temperatures (up to 800 °C) was noticeably increased due to the addition of nanosilica. Similar conclusions were also drawn by Horszczaruk et al. [71], who analyzed the effect of temperatures, which ranged from 20 °C to 800 °C, on the thermal resistance of cement mortar incorporating nanosilica (in the amount from 1 wt% to 5 wt%). Both SEM and optical microscopy observations showed that, up to 3 wt%, the inclusion of nanosilica can produce additional C–S–H through the pozzolanic reaction, which contributed to the improvement in the microstructure and exhibited the ability of bridging cracks after exposure to elevated temperatures. Hence, the admixed nanosilica improved the thermal resistance of cement mortar, especially at temperatures up to 200 °C.

Heikal et al. [70] reported that the incorporation of 1 wt% nano-Al_2_O_3_ has an accelerating effect on the hydration of cement paste through acting as a nanofiller, thus resulting in a better firing resistance up to 1000 °C than other pastes. The densification and compaction of the microstructure due to the addition of nano-Al_2_O_3_, have also been observed. Irshidat and Al-Saleh [72] investigated the effect of the addition of nanoclay on the thermal performance of the cement mortar, with the study showing higher residual compressive strength at 200 °C and higher residual flexural and tensile strengths at 400 °C, when the optimal dosage of nanoclay (2% by weight of cement) was admixed to modify the mortar. The presence of nanoclay was observed to cause the reduction in the density and width of the hairline cracks generated during the elevated temperature exposure.

**Figure 7:**
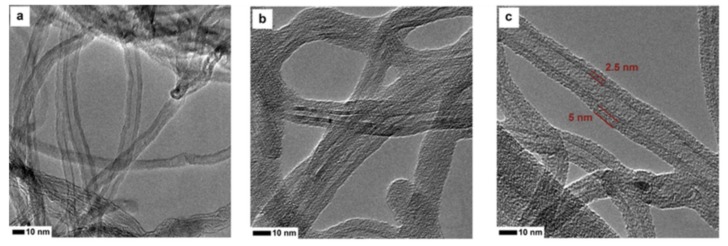
TEM images of (**a**) CNT and (**b**,**c**) CNT/nanosilica. (The average thickness of the shell of nanosilica is 5 nm). Adapted from [14], with permission from Elsevier, 2019.

Reducing the cost of nanomaterial production was taken into consideration in a study conducted by Sikora et al. [14], in which the authors synthesized both CNT and nanosilica from recycled substrates Specifically, they prepared the CNT/nanosilica core/shell structures and analyzed the effects of the elevated temperature on the cement pastes containing the obtained core–shell nanostructure. Transmission electron microscope (TEM) observations confirmed that the surfaces of CNT were successfully covered by a shell of nanosilica (Figure 7), which can not only improve the bond between the CNT and cement paste but also protect the CNT from calcination during heating, resulting in an extended temperature range that CNT can exhibit positive influence on the paste. Hence, the samples containing CNT/nanosilica exhibited compressive strength retention up to 600 °C, while pristine CNT-incorporated samples exhibited gradual strength loss after exposure to 450 °C. Studies undertaken by Zhang et al. [76] and Amin et al. [77] have reported that when CNT is incorporated in cementitious composites, it does not improve or impede the cement hydration but can act as a channel to assist in the release of high-pressure steam caused by high temperatures or work as bridges between hydration products and cracks. Mohammed et al. [74] analyzed the effect of GO on the high-temperature performance of concrete and suggested that, due to modification of the pore structure by GO, the amount of capillary pores was reduced and the amount of gel pores was increased, resulting in a more compatible thermal deformation and better cracking resistance. As a result, the residual compressive strength of the GO-modified samples was 70% of the original value, while the counterpart for the reference samples was only 35% after being exposed to high temperatures. In addition to GO, GSNS, another type derivative of graphene, was investigated for its effect on the mechanical properties of concrete during high temperature exposure [68]. The study found that the sulfonic groups in GSNS can participate in the reaction with hydration products, resulting in covalent bonding between GSNS and matrix. Therefore, an enhanced residual strength of concrete during exposure to temperatures up to 1000 °C can be achieved, as a result of the improved microstructure.

## 5. Nanotechnology in Reinforced Concrete

Corrosion of the reinforcing streel in concrete is considered a significant contributor to deficiencies in reinforced concrete (RC), which result in (often premature) failure of civil infrastructure in the United States and worldwide [78]. Generally, the embedded steel is protected by the alkaline solution in the pores of the concrete, through formation of a passive oxide/hydroxide layer on the surface of steel rebar [79]. However, this passivation of steel rebar can be disrupted by the reduction in alkalinity (due to carbonation or acid attack) or the presence of excessive chloride ions [78]. For instance, when exposed to chloride anions at a concentration higher than the depassivation threshold, the Cl-containing parts in the oxide film on rebar are destroyed and washed away, forming active pits, which act as corrosion initiation sites [80]. The corrosion products are expansive and thereby can cause the cracking and spalling in concrete. This, coupled with the reduction in the cross-section area of the rebar, could result in the highly unpredictable failure of RC structures [78]. In order to mitigate the corrosion of steel rebar, several basic approaches are widely used: improving the transport properties of the embedding concrete, use of a coating for reinforced steel, and cathodic protection [81]. Recently, nanotechnology has been employed in combination with the conventional countermeasures of rebar corrosion to enhance the long-term durability of RC in corrosive environments.

### 5.1. Nanomaterials Addition

The admixing of nanomaterials, including nanosilica, nano-CaCO_3_, CNT, and carbon nanofiber, can increase the resistance of the CBMS to rebar corrosion [82,83,84,85,86]. The presence of nanosilica and nano-CaCO_3_ in fly ash concretes decreases the total capillary porosity and the diameter of pores, thus reducing the water permeability and chloride diffusivity, and ultimately lowering the corrosion rate of the embedded rebar [82]. It should be noted that although the addition of the nanosilica or nano-CaCO_3_ could reduce the amount of calcium hydroxide present in the fly ash concrete and thereby reduce the alkalinity of the concrete pore solution, the benefits of nanomodification to the transport properties of the concrete were more significant. The overall result was thus mitigated rebar corrosion by chlorides. Another study also reported that the inclusion of nanosilica in concrete could delay the initiation of corrosion and lower the corrosion rate indicated by increased polarization resistance of steel rebar in concrete [86]. Furthermore, the incorporation of CNT and carbon nanofiber in the reinforced cement composite delays the onset of active corrosion of rebar and reduces the corrosion rate of rebar, which results from the reduced porosity of the matrix and the controlled coalescing process of cracking [83]. 

**Figure 8:**
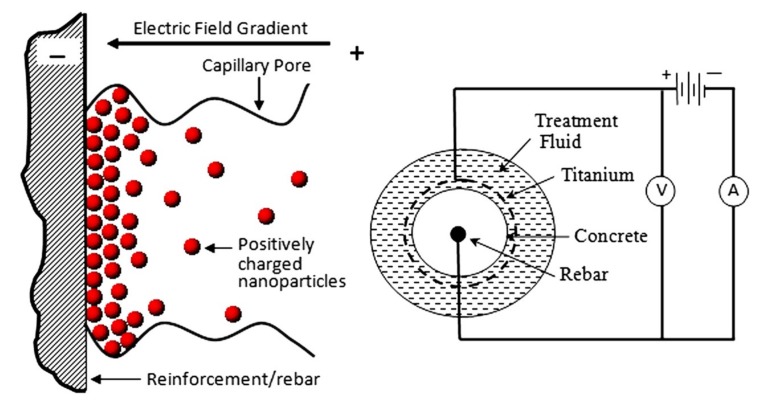
Concept of the nanoparticles transport into the capillary pores and the electrokinetic nanoparticles treatment circuit. Reproduced from [87], with permission from American Society of Civil Engineers, 2019.

Other than serving as admixtures in fresh concrete, nanoparticles have been also explored for their application in rehabilitation of old concrete. A novel method, named the electrokinetic nanoparticles (EN) treatment, is designed to inject the electrically charged nanomaterials (nanosilica and nanoalumina) into aged concrete and move them towards the reinforced rebar by applying an electric field, as illustrated in Figure 8 [87]. In addition to rehabilitating the cracked concrete, this treatment is also effective in mitigating the reinforcement corrosion, which can be attributed to the reduced chloride content, improved microstructure, and additionally generated C–S–H. 

### 5.2. Nanomaterials Coating

**Figure 9:**
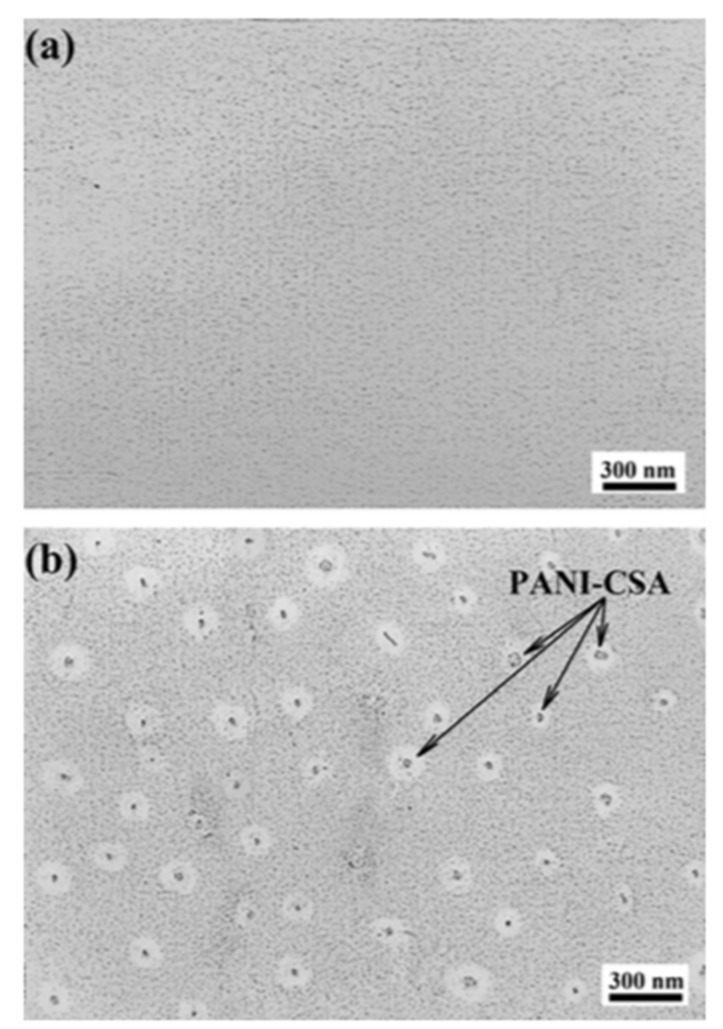
TEM images of (**a**) epoxy and (**b**) epoxy/polyaniline–camphor sulfonate (PANI-CSA) nanocomposite coatings. Reproduced from [79], with permission from Elsevier, 2019.

As a coating material, epoxy has been widely used to protect the steel rebar in RC from corroding and to improve the anticorrosive performance of the steel reinforcement. However, due to the porous and hydrophilic nature of epoxy, it cannot protect the steel reinforcement for a long term [79,88]. Adding nanomaterials into epoxy is a feasible approach to improve the performance of epoxy as a coating on the steel rebar. One study first dispersed the polyaniline–camphor sulfonate (PANI-CSA) in epoxy and then coated the steel rebar with the obtained homogeneous epoxy/PANI-CSA self-healing nanocomposite (Figure 9). The coated steel rebar still exhibited a protective passive layer even after the corresponding concrete was exposed in the chloride-laden environment up to one year [79]. In comparison, the epoxy-coated rebars only showed a similar performance within 130 days. The improvement of nanomodified coating was attributed to the uniformly distributed nanoparticles in the epoxy, making it a more effective coating against the ingress of detrimental species. Similar studies indicated that the epoxy coating that contains nano-Fe_2_O_3_, halloysite nano clay, or nano-ZrO_2_ can significantly improve the corrosion resistance of the coated steel immersed in a NaCl solution [88,89]. An enhanced coating barrier, which results from the addition of nanomaterials and makes water and ionic species difficult to transport, was thought to mainly account for this improvement in the anticorrosive performance of the epoxy coating [88].

## 6. Nanoscale Modeling in CBMS

In the context of engineering, concrete is a heterogeneous composite material in which aggregates are surrounded by cement paste. The cement paste is a porous solid composite which mainly contains semicrystalline C–S–H together with calcium hydrates [90]. So far, the nanostructure of C–S–H has been extensively studied, with the suggested models ranging from colloidal to “layer-like”. Several crystalline structures, including Tobermorite, Jennite, Clinotobermorite, and Foshagite, have been also reported to simulate the structure of C–S–H [2,91,92,93,94,95]. Furthermore, the C–S–H that features low density or high density tends to be generated in the cement hydrates under different curing conditions and with or without specific nanomaterials [96,97]. Zhu et al. [98] demonstrated that the addition of nano-SnO_2_ can promote the generation of high-density C–S–H and reduce the amount of low-density C–S–H found in cement composites. With the addition of nanomaterials, there has been increasing interest in the nanoscale modeling of the hydration process and the interaction between the admixed nanomaterials and hydration products in CBMS. For instance, Liu and Shi [99] conducted molecular dynamics (MD) simulations to computationally investigate the nanoscale interactions between NaNO_2_ (corrosion inhibiting admixture), water molecules, nanoparticles (Al_2_O_3_, Fe_2_O_3_, SiO_2_, and TiO_2_), and representative minerals in hydrated cement (ettringite, Friedel’s salt, jennite, kuzelite, portlandite, and tobermorite).

Nanosilica in cement composite can react with Ca(OH)_2_ to generate additional C–S–H, which exhibits higher rigidity than the one that forms in the pure cement paste [100]. In fact, the volume fraction of high-stiffness C–S–H can be as high as 50% in the cement composite containing nanosilica [101]. Moreover, the average chain length of C–S–H gel is also increased by the addition of nanosilica [101,102].

**Figure 10:**
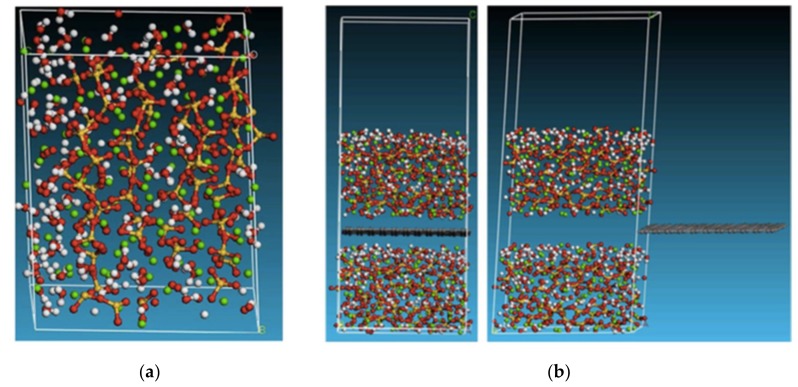
Atomic model for (**a**) calcium–silicate–hydrates (C–S–H) and (**b**) graphene C–S–H hybrid nanocomposite interfacial pullout. Reproduced from [103], with permission from American Society of Civil Engineers, 2019.

Featuring an isolated individual sheet structure, graphene and GO have been added into the cement composite to study the interaction mechanisms between C–S–H and the admixed graphene or GO [103,104,105,106]. Alkhteb et al. [103] investigated the interfacial strength between C–S–H and functionalized graphene nanoplatelets in cement composites. In their work, a molecular structure of C–S–H that features short silica chains was adopted to reflect the realistic values of Ca/Si ratio and density of C–S–H (Figure 10a). Figure 10b shows the atomic model for the interface between the proposed C–S–H and graphene in the pullout test. Test results indicated that the functionalized graphene improved the interfacial strength and thus increased the overall mechanical properties of the cement composites. 

**Figure 11:**
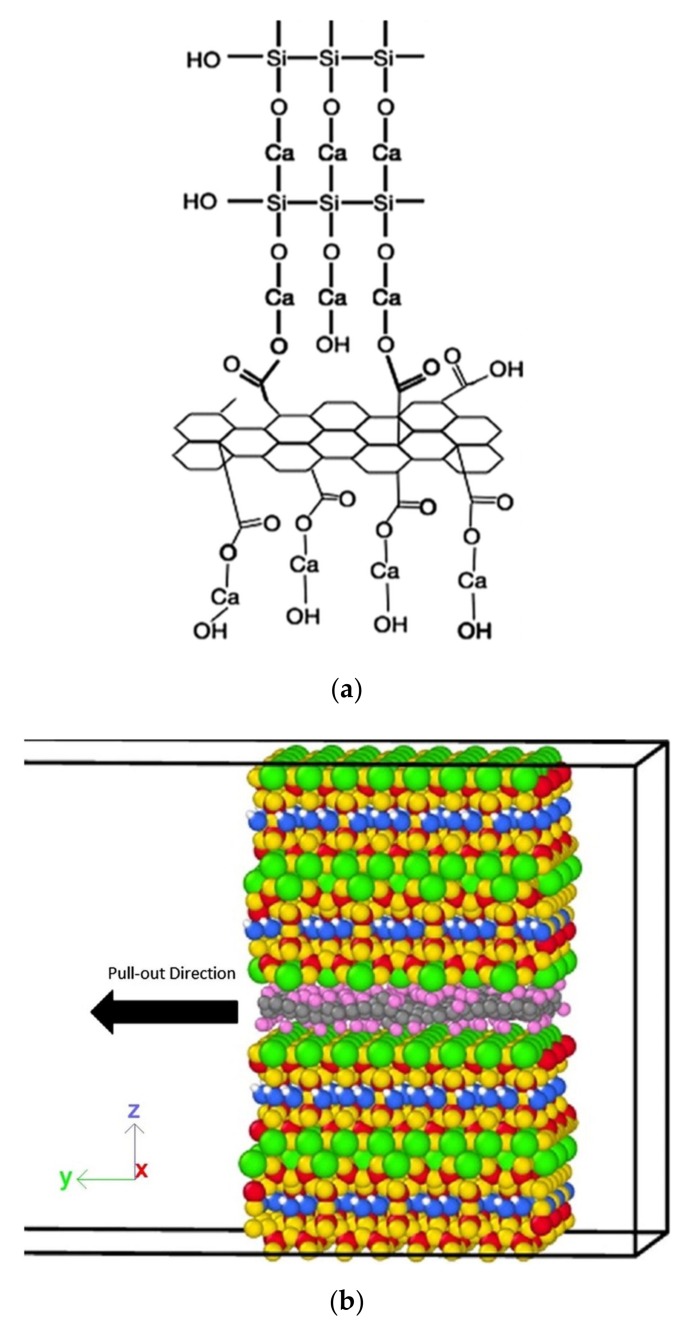
Illustration of (**a**) graphene oxide (GO)/C–S–H composite and (**b**) pull-out simulation at the nanoscale. Reproduced from [104], with permission from Elsevier, 2019.

Fan et al. [104] modeled the interface between C–S–H and GO and studied the stress transferring mechanism. They used the tobermorite structure as the base structure of C–S–H, while the GO structure featured functional groups (including epoxy and hydroxyl) distributed randomly on the carbon plane. With these assumptions, the nanostructure of the C–S–H containing GO can be illustrated in Figure 11a, in which an interface exists resulting from the reaction between the oxygen atoms of the GO sheet and the calcium atoms from the C–S–H. Pull-out tests (Figure 11b) were carried out in a realistic manner and the results showed that the shear strength of the GO/C–S–H interface can be as high as 647 MPa, which indicated a strong interfacial bonding strength between the GO and C–S–H. 

**Figure 12:**
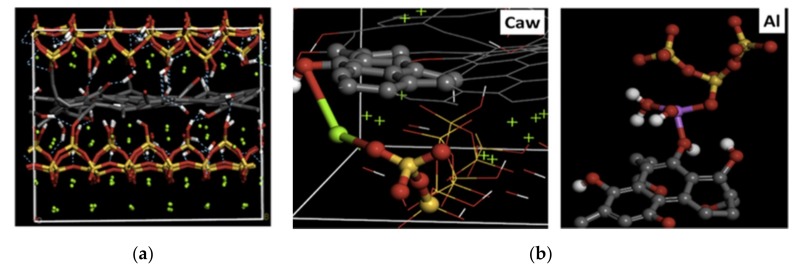
Molecular structure of the (**a**) GO/C–S–H; (**b**) oxygen–calcium–oxygen bond (O–Ca–O) and oxygen–aluminum–oxygen bond (O–Al–O) connecting the neighboring GO and C–S–H. The ball-stick styles represent the composite model. Oxygen, calcium, carbon, silicon, hydrogen, and aluminum atoms are represented by red, green, gray, yellow, white, and purple balls, respectively. The hydroxyl, C–C, and silicate bonds are represented by white-red, gray, and yellow-red sticks, respectively. Reproduced from [105], with permission from Elsevier, 2019.

Hou et al. [105] also simulated the C–S–H substrate based on the tobermorite structure and studied the interaction mechanisms between GO and cement hydration products. It was found that the functional hydroxyl groups in GO can accept hydrogen-bonds of interlayer water molecules in the C–S–H (Figure 12a). Additionally, the Ca^2+^ and Al^3+^ ions present in the hydrates can bridge the oxygen atoms in silicate chains and hydroxyl groups in GO, forming a longer silicate chain and facilitating the crack-bridging effect of GO, as shown in Figure 12b. A recent study conducted by Hou et al. [106] has indicated that the hydroxyl and carboxyl groups in GO can act as oxygen sites to connect the H-bond and neighboring ions, resulting in an improved resistance to the transport of fluid in gel pores. Moreover, the carboxyl groups in GO exhibited the ability to root on the C–S–H deeply, which further impedes the connectivity of the transport channels for water molecules and ions.

**Figure 13:**
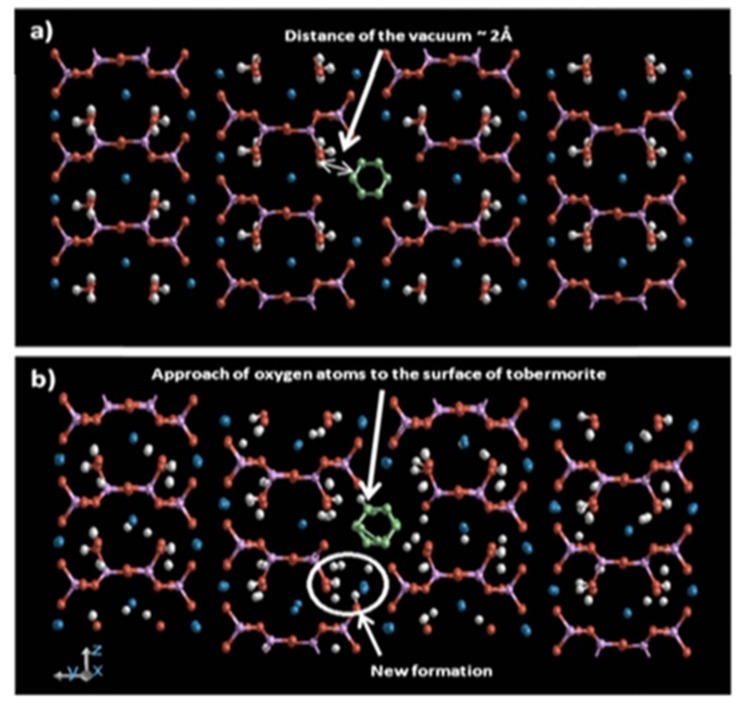
Snapshot of the CNT/C–S–H system: (**a**) CNT’s insertion into the hole of C–S–H and (**b**) formation of new morphology. Reproduced from [95], with permission from Elsevier, 2019.

Simulating C–S–H gel with the most used Tobermorite model, Lushnikova and Zaoui [95] inserted CNT into the hole in C–S–H and studied the improvement mechanism. Figure 13 shows the interaction between the inserted CNT and the C–S–H. CNT was located at a position where the CNT was kept at a distance of 2 Å away from the surrounding C–S–H gel. This distance was observed to allow the oxygen atoms in the C–S–H be attracted to the structure of CNT, contributing to the generation of new morphology. These newly formed products, along with the admixed CNT, gave the C–S–H improved mechanical properties, such as increased bulk modulus, shear modulus, plane stress, and Young’s modulus, thereby improving the performance of the cement/CNT composites. With the aid of finite element method, a multiscale modeling approach can be used to evaluate the nonlinear constitutive behavior of the CNT reinforced concrete from the nanoscale to the mesoscale [107]. In this proposed model, the structure of C–S–H and the porosity can be considered as parameters to reflect any changing nanoscale characteristics of concrete at the nanoscale.

## 7. Characterization of CBMS at Nanoscale

The performance of CBMS is strongly influenced by the properties of their micro- and nanostructures [108]. Alteration of these microscopic structures of CBMS due to the addition of nanomaterials raises the need for special instruments and methodologies to characterize the resulting nanocomposites and interfaces. This section presents the advanced methods employed to characterize the properties, chemistry, and morphology of CBMS at the nanoscale. 

### 7.1. Mechanical Characterization

One powerful technique used to assess the mechanical properties of cementitious materials at the nanoscale is nanoindentation [109]. Nanoindentation is conducted by introducing a gradually-increasing force via the indenter tip. When the tip penetrates to a certain depth, the unloading phase initiates until the sample is completely unloaded. Several material properties can be obtained by the nanoindentation test, such as hardness and elastic modulus [110,111]. Hu et al. [112] used nanoindentation to show that the addition of CNT at 0.2 wt% increased the fraction of dense C–S–H in the cement matrix and increased the composite’s compressive strength. Nanoindentation also enabled researchers to identify two types of C–S–H existing in the hardened cement paste [108]. The mechanical properties of C–S–H with low density was significantly affected by calcium leaching, unlike the dense C–S–H zone where the calcium leaching effect was negligible. Moreover, while the macroscopic creep properties of cementitious material can only be caused by long-term loading, nanoindentation is capable of capturing representative creep properties of cementitious material in minutes. Researchers hypothesized that nanoindentation facilitates the application of much higher stresses than the macroscopic creep experiment [113]. Nevertheless, researchers have reported several difficulties associated with conducting nanoindentation, such as producing samples with a flat surface to ensure a constant indentation depth. In addition, the samples must be preserved in a vacuum storage to prevent carbonation due to the airborne CO_2_, which is difficult to ensure [1]. 

### 7.2. C–S–H Structure Characterization

Characterization of the structure of C–S–H at the nanoscale is crucial to understand and then enhance the behavior and properties of CBMS at the macroscale. To investigate the structure of hydration products at the nanoscale, several tools are used, including the nuclear magnetic resonance (NMR). NMR provides an insight on the nanostructure of C–S–H in hydration products. A typical NMR spectrograph presents the type and relative amount of bonds that formed in the material for the scanned atom. In the C–S–H characterization, silicon ^29^Si is typically used and the bonding is reported as Q^n^ where n represents the number of oxygen-bonded silicon atoms to one silicon atom [114,115,116]. The n value ranges between zero for the free silicon atom and four for the fully polymerized silicon [113]. Silicon bonds are influenced by the interlayers water content and hence, NMR can be used indirectly for identifying the interlayers water content in C–S–H samples [114]. The evolution of C–S–H gel can also be monitored by NMR to provide a comprehensive understanding of the hydration process at different ages. The ^29^Si NMR spectra of the hydration of tricalcium silicate (C_3_S) in the dilute system revealed that Q^0^ was decreasing steadily, which reflects a continuous increase in the hydration degree of C_3_S [117]. Conversely, Q^2^ showed a continuous increase over time, reflecting the formation of long silicate chains. In addition, Bae et al. [118] used ^29^Si and ^27^Al NMR to analyze the chemical composition of hydration products of the C_3_S-high volume fly ash (HVFA) system. Results showed that the C–S–H resulting from the C_3_S-HVFA system experienced higher degree of silicate polymerization. Specifically, the silicate chain length was four times longer than the corresponding C–S–H resulting from the pure C_3_S system. ^27^Al NMR spectra confirmed the presence of Al in the C–S–H resulting from the C_3_S-HVFA system, where Al substituted Si at the C–S–H bridging tetrahedral sites. Xu et al. [24] employed both ^29^Si and ^27^Al NMR to shed light on the chemical bonds of cement pastes incorporating 0.02 wt% GO. Along with other characterization results, the NMR examination suggested that “GO can attract Ca cations to produce jennite-like hydrates near the GO nanosheets” and “increase the polymerization of the hydrates”.

Another widely used technique to explore micro- and nanostructures of hydration products is the small angle neutron scattering (SANS). A basic SANS experiment is conducted by sending a neutron beam to the sample and measuring the small-angle scattering intensity on a two-dimensions detector [119,120,121]. Using SANS, Chiang et al. [122] developed an analytical model that describes the structures of C–S–H. SANS can also be used to investigate the influence of CaCl_2_ on the hydration kinetics and the nanostructure of cement paste [120]. Results indicated that CaCl_2_ accelerated the hydration process, while it also increased the drying shrinkage of cement paste samples.

Quasielastic neutron scattering (QENS) is yet another advanced tool used in the characterization of materials at the nanoscale. Unlike SANS, QENS is one type of inelastic neutron scattering where the neutron beam is scattered due to dynamic interaction with the sample. Li et al. [123] investigated the state of water existing in the C–S–H gel with different water contents using QENS. Results were valuable in describing the transport regime of water in the C–S–H gel pores, which was in correlation with the water content and temperature. 

### 7.3. Imaging Characterization

It is crucial to have a clear vision of the interactions between nanomaterials and hydration products at the nanoscale in order to obtain a clearer understanding of the nanomodification mechanism in CBMS. This section presents several imaging techniques that utilize different approaches to deliver high resolution images at the nanoscale. 

**Figure 14:**
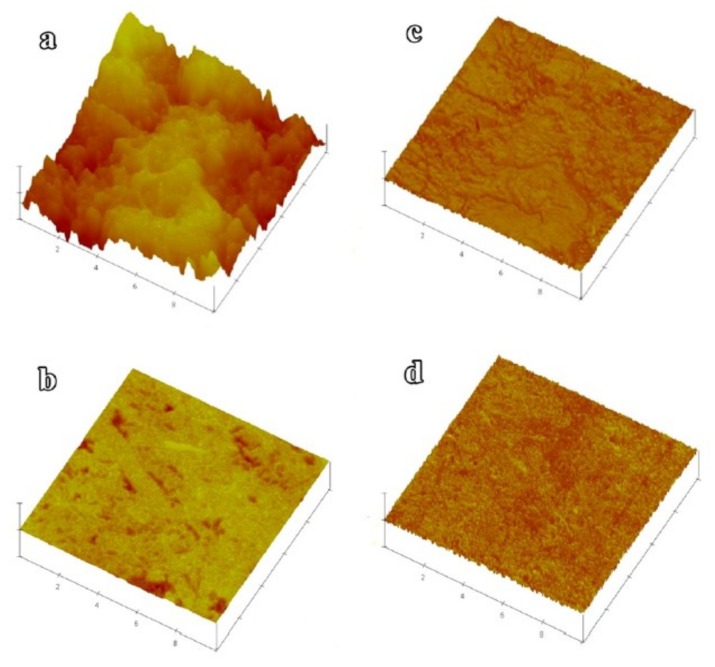
Atomic force microscope images of (**a**) height image of the conventional cement paste, (**b**) height image of the cement paste mixed with nano-TiO_2_, (**c**) phase image of the conventional cement paste, (**d**) phase image of the cement paste mixed with nano-TiO_2_. Reproduced from [131], with permission from American Chemical Society, 2019.

The atomic force microscope (AFM) is widely used as an imaging tool at the nanoscale. AFM operates by applying force on the material, by using a sharp probe which is fixed on a cantilever arm [124]. By measuring the vertical and lateral deflection of the cantilever arm via an optical lever, the image is constructed [125,126,127]. AFM facilitated the investigation of the dispersion of MWCNT in the cement paste [128]. Furthermore, AFM can be used to characterize the morphology of the Portland cement paste containing graphene nanoplatelets at the early age of the hydration process [103]. AFM can also be used to investigate the nanoroughness of cement paste samples with admixed SiO_2_ nanoparticles. It was illustrated that the addition of nano-SiO_2_ increased the surface roughness of the hardened cement paste and facilitated the formation of larger particles of C–S–H in the cement paste [129,130]. However, the AFM results revealed that the cement paste with admixed nano-TiO_2_ showed much lower nanoroughness than the reference cement paste (Figure 14) [131]. Researchers have pointed out that AFM has a relatively long scanning time which can be considered as a drawback [132]. 

**Figure 15:**
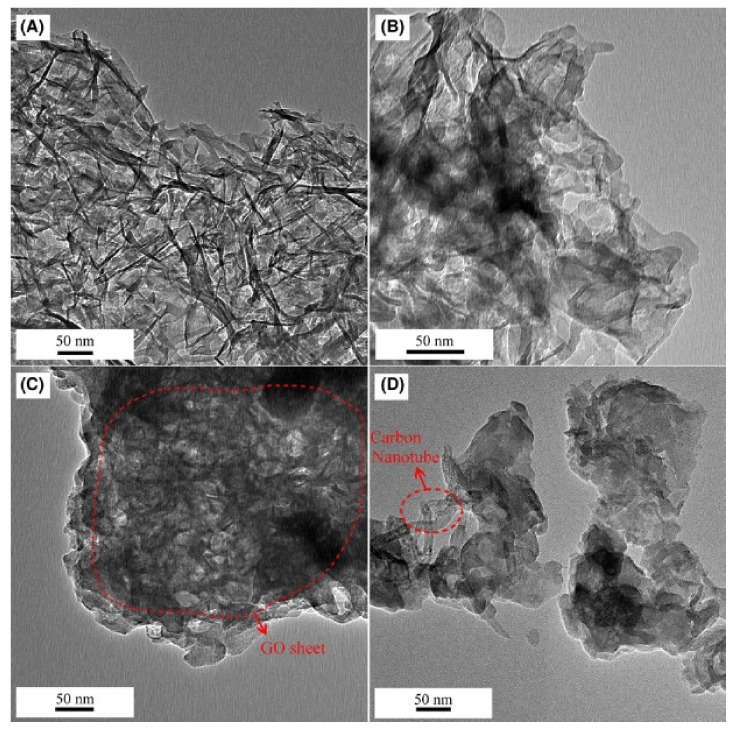
TEM images of the C–S–H incorporate different nanomaterials: (**A**) 2% nano-TiO_2_, (**B**) 2% nano-SiO_2_, (**C**) 0.5% GO, (**D**) 0.5% CNT. Reproduced from [129], with permission from John Wiley and Sons, 2019.

Another technique that offers high-resolution images of the material’s nanostructure is the transmission electron microscopy (TEM). Unlike AFM, TEM operates by transmitting a beam of electrons to penetrate through the material and construct an image [133,134]. It should be noted that images obtained only from TEM are not reliable to characterize the tested materials, and hence, TEM results are usually coupled with data from other characterization techniques for better results interpretation [1]. TEM was used to explore the effect of curing temperature on the chemical and mechanical properties of the oil-well cement paste with a high silica content [135]. Generally, neither of the paste samples cured at 200 °C or 175 °C exhibited a crystalline morphology in the C–S–H. However, Li et al. [129] found that the addition of nano-TiO_2_, nano-SiO_2_, CNT, and GO to the cement paste yielded a C–S–H with more crystalline nanostructure (i.e., short range order), as illustrated by the TEM images in Figure 15.

**Figure 16:**
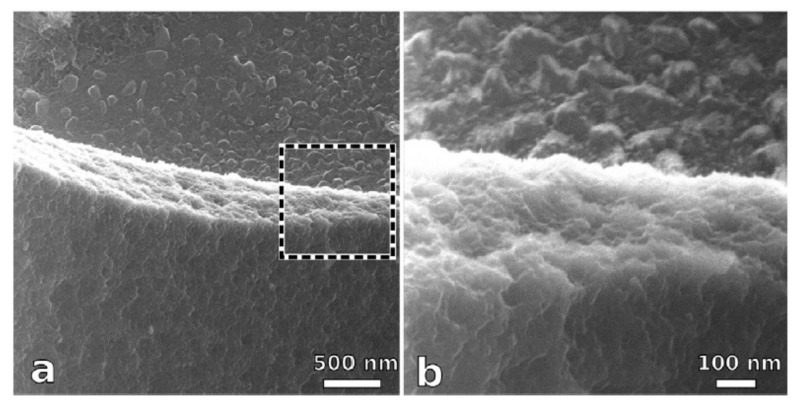
Helium ion microscopy (HIM) image of a foil-like C–A–S–H gel covering a ground-granulated blast furnace slag (GGBFS) particle: (**a**) low magnification and (**b**) high magnification of the selected area in (a). The background shows globules of the C–A–S–H gel. Reproduced from [138], with permission from Elsevier, 2019.

The helium ion microscopy (HIM) is another advanced imaging tool that can be used to examine hydration products at the nanoscale. HIM operates similar to TEM, except that electrons are replaced by Helium ions which induce less scattering when they infiltrate the solid bodies. Consequently, the imaging resolution is highly improved [136,137]. HIM was applied on a sample of alkali-activated ground-granulated blast furnace slag (GGBFS) cement paste to explore the morphology of this material at micro- and nanoscales [138]. The high-resolution images resulting from HIM revealed two types of heterogeneous calcium–aluminate–silicate–hydrate (C–A–S–H) gel in the paste. The inner product of the C–A–S–H gel showed a foil-like morphology whereas the outer product possesses a spherical morphology, as shown in Figure 16. However, there are several drawbacks of HIM reported, such as high cost and relatively low material contrast, which make it difficult to detect the edges of the material phases [139]. 

### 7.4. Pore Structure Characterization

The pore structure of hydration products at the nanoscale contributes significantly to the durability and service life of CBMS such as concrete [140,141]. One of the common methods of characterizing the pore structure in the porous media is mercury intrusion porosimetry (MIP), which is simply applied by pressurizing a nonwetting liquid, mercury, into the porous media [142,143]. In order to study the pore structures at the nanoscale, a high pressure is required to drive the liquid mercury inside pores. To capture pores at a minimum size of 3 nm, the maximum applied pressure can be as high as 414 MPa [144]. Furthermore, MIP can be used to study the average pore diameter and pore size distribution of cement pastes incorporating nano-TiO_2_ and nano-SiO_2_ [145]. In comparison with conventional cement paste, the addition of nanoparticles was found to reduce the average pore size to the range of 20–50 nm, which is considered adequate for resisting chloride ingress. Although MIP is widely used as a pore characterization technique, researchers have reported several drawbacks such as the failure of MIP to detect isolated pores and the high pressure required to detect gel pores of C–S–H in hydration products [142].

Another valuable nondestructive tool that can be used to characterize the pore network of hydration products at the nanoscale is the nano x-ray computed tomography (nano-CT), which uses a stack of x-ray images to build the three-dimensional (3D) image of the tested sample [146]. Using nano-CT to explore the pore network in a leached cement paste sample, it was illustrated that the pore connectivity at the nanoscale is much higher than that at the micro scale [147]. Wang and Dai [146] used nano-CT to obtain critical information on the pore structure of the cement paste sample, such as pore volume, connectivity, and permeability, which can be used in concrete service life prediction models. Nevertheless, the image resolution can be affected by the radiation damage and unstable sample positioning which consequently affect the constructed 3D images [148,149]. 

**Figure 17:**
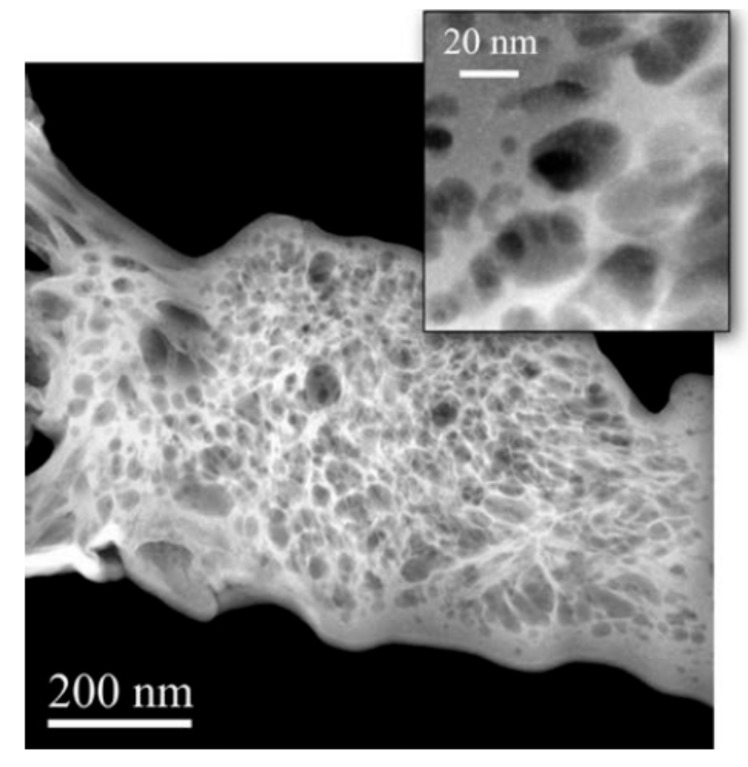
TEM image of the pore network in the C–S–H. Reproduced from [150], with permission from Elsevier, 2019.

Wenzel et al. [150] modified TEM using the focused ion beam (FIB) technique to visualize the pore structure of the hardened cement paste. The resulted images revealed a honeycomb structure of gel pores with a diameter up to 50 nm (Figure 17). Although the TEM–FIB technique provides a valuable understanding of the pore structure of hydration products, it does not provide a quantitative information on the characteristics of the pore structure of the cement composite. 

## 8. Concluding Remarks

Nanotechnology is an effective approach to improve the durability performances of cement-based materials. This work provides a comprehensive overview with regard to the current knowledge on multiple dimensions of the effect of nanomaterials on CBMS, based mainly on the papers published over the last decade. Some key findings from the review are concluded as follows.

- Dispersion of nanomaterials in CBMS plays an important role in ensuring the effectiveness of nanomaterials to mitigate the deteriorations of cement composites. Common applied approaches to disperse nanomaterials include the use of surfactant, application of ultrasonication, and functionalization of nanomaterials.
- Physical deteriorations of CBMS including shrinkage, freeze–thaw damage, and abrasion can be reduced by the admixed nanomaterials, resulting from a denser and less permeable mixture.
- Adding the optimal type and dosage of nanomaterials is an effective approach to improve the resistance of CBMS to chemical deteriorations, such as sulfate attack, acid attack, alkali-aggregate reactions, and thermal degradation.
- Admixing nanomaterials in fresh concrete, electrically injecting nanomaterials into aged concrete, and coating the rebar with nanomodified epoxy coating are among practical and effective approaches to improve the resistance of reinforced concrete against rebar corrosion.
- Modeling of the interactions between C–S–H gel and the admixed nanomaterials can facilitate a mechanistic understanding of the relationship between the nanostructure and the properties of CBMS.
- Nanoindentation is a powerful technique used to characterize the nanoscale mechanical properties of cement composite, while MIP is usually suitable for characterization of the pore structure. The most adopted techniques for the nanoscale imaging and C–S–H structure characterizations of CBMS are AFM, TEM, and HIM, and NMR, SANS, and QENS, respectively.

There are some knowledge gaps or remaining challenges that need to be addressed before widespread adoption of nanomaterials in the construction practices.

- A gap exists between the academic research at the laboratory scale and the realistic engineering applications. It is important to adapt the nanotechnology to meet the requirements and constraints of the traditional practices generally adopted by the construction industry [2].
- The cost-effectiveness of nanomaterials must be evaluated before their use in CBMS, and this should be done from a life-cycle perspective. Despite the seemingly high initial cost of most nanomaterials, nanomaterials can greatly improve the durability and service life of CBMS, resulting in much lower costs during the use phase of CBMS (e.g., those for monitoring, maintenance, and repair). Nanotechnology will likely result in a competitive life-cycle cost for many constructions using CBMS.
- Although nanotechnology exhibits the potential for great innovations in the construction industry, potential health risks should be assessed and addressed for practitioners to ensure appropriate use of nanomaterials by the industry.

Looking to the future, nanotechnology will continually play a powerful role in advancing cement and concrete technology and unlocking the potential of conventional and unconventional cementitious materials. It will help to establish the aforementioned fundamental understanding of the effect of nanomaterials in CBMS from the bottom up, through the use of multiscale modeling of the hydration process, rheological behavior and deterioration processes, and through the use of advanced characterization methods (e.g., TEM, SAXS, NMR, and nanoindentation). Furthermore, the addition of nanomaterials (e.g., modification by nanosilica, graphene oxide, and carbon nanofiber) will enable the regulation and manipulation of hydration products and their microstructure in cement-based materials, to achieve their desirable properties and life-cycle performances.
